# Genetically predicted blood metabolites mediate relationships between gut microbiota and ovarian cancer: a Mendelian randomization study

**DOI:** 10.3389/fcimb.2024.1451880

**Published:** 2024-09-19

**Authors:** Liang Zhang, Tao Cao, Kang Liu, Pengyu Sun, Wenhao Wang, Jiani Guo

**Affiliations:** ^1^ Department of Obstetrics and Gynecology, Shanxi Bethune Hospital and Shanxi Academy of Medical Sciences, Shanxi Bethune Hospital Affiliated to Shanxi Medical University, Taiyuan, China; ^2^ Department of Obstetrics and Gynecology, Second Hospital of Shanxi Medical University, Taiyuan, China; ^3^ Department of Critical Care Medicine, Shanxi Bethune Hospital and Shanxi Academy of Medical Sciences, Shanxi Bethune Hospital Affiliated to Shanxi Medical University, Taiyuan, China

**Keywords:** gut microbiota (GM), ovarian cancer (OC), metabolites, Mendelian randomization (MR) analysis, biomarker

## Abstract

**Background and purpose:**

While there is evidence that gut microbiota (GM) and blood metabolites are associated with ovarian cancer (OC), the precise mechanisms underlying this relationship are still unclear. This study used Mendelian randomization (MR) to elucidate the causal connections between GM, blood metabolite biomarkers, and OC.

**Methods:**

In this study, we leveraged summary data for GM (5,959 individuals with genotype-matched GM), blood metabolites (233 circulating metabolic traits with 136,016 participants), and OC (63,702 participants with 23,564 cases and 40,138 controls) from genome-wide association studies (GWASs). We performed MR analysis to explore the causal relationship between GM and OC. Further, we harnessed univariable MR (UVMR) analysis to evaluate the causal associations between GM and circulating metabolites. Finally, we employed a two-step approach based on multivariable MR (MVMR) to evaluate the total genetic prediction effect of metabolites mediating the GM on the risk of OC to discover a potential causal relationship.

**Results:**

In the MR analysis, 24 gut bacteria were causally associated with the pathogenesis of OC, including 10 gut bacteria (*Dorea phocaeense*, *Succinivibrionaceae*, *Raoultella*, *Phascolarctobacterium sp003150755*, *Paenibacillus J*, *NK4A144*, *K10*, *UCG-010 sp003150215*, *Pseudomonas aeruginosa*, and *Planococcaceae*) that were risk factors, and 14 gut bacteria (*CAG-177 sp002438685*, *GCA-900066135 sp900066135*, *Enorma massiliensis*, *Odoribacter laneus*, *Ruminococcus E sp003521625*, *Streptococcus sanguinis*, *Turicibacter sp001543345*, *Bacillus velezensis*, *CAG-977*, *CyanobacteriaStaphylococcus A fleurettii*, *Caloranaerobacteraceae*, *RUG472 sp900319345*, and *CAG-269 sp001915995*) that were protective factors. The UVMR analysis showed that these 24 positive gut bacteria were causally related to lipoproteins, lipids, and amino acids. According to the MVMR analysis, *Enorma massiliensis* could reduce the risk of OC by raising the total cholesterol to total lipids ratio in large low-density lipoprotein (LDL) and cholesteryl esters to total lipids ratio in intermediate-density lipoprotein (IDL). *Turicibacter sp001543345*, however, could reduce the risk of OC by lowering free cholesterol in small high-density lipoprotein (HDL) and increasing the ratios of saturated fatty acids to total fatty acids, total cholesterol to total lipids ratio in very small very-low-density lipoprotein (VLDL), and cholesteryl esters to total lipids ratio in very small VLDL.

**Conclusion:**

The current MR study provides evidence that genetically predicted blood metabolites can mediate relationships between GM and OC.

## 1 Introduction

Ovarian cancer (OC) is a prevalent type of cancer affecting women worldwide and with a high incidence and the lowest survival rate in all gynecological malignancies, is seriously endangering women’s health (Salehi et al., 2019; Sung et al., 2021; Yang et al., 2022; Zara et al., 2022). The early symptoms of OC are imperceptible, and most of them are already advanced when diagnosed (Torre et al., 2018). It is very significant to clarify the incidence factors for the treatment and prevention of OC; however, the pathogenesis of OC is still unknown.

Studies have shown that the risk factors for OC mainly include family history of OC or endometriosis, environmental pollution, and bad living habits (Mallen et al., 2018). Observational and experimental studies have recently shown an association between gut microbial dysbiosis and the occurrence of various tumors, including gastric, breast, and intestinal cancer (Banerjee et al., 2017; Łaniewski et al., 2020). Patients with OC are sensitive to the gut microbiota (GM), often showing obvious intestinal symptoms in the early stage of the onset, including abdominal pain and distension, indigestion, constipation, and early satiety. Moreover, the gastrointestinal symptoms of patients with OC are more prominent in the treatment process than those of patients with cervical cancer or endometrial cancer. Some scholars have compared and analyzed the GM in high-grade serous OC (HGSOC) and benign tumors by 16S rRNA sequencing and confirmed that gut microbial dysbiosis played an important role in OC with animal models (Zhou et al., 2019; Hu et al., 2023). Furthermore, GM are associated with chemotherapy sensitivity, and the regulation of GM can alleviate cisplatin resistance in OC (Chambers et al., 2022), while fecal microbiota transplantation of *Akkermansia muciniphila* plays an important role in inhibiting OC progression through T cell activation (Wang et al., 2022). In addition, the proportion of Proteobacteria and Firmicutes was significantly higher in cancer samples than in controls. In a 2019 study, Nené et al. reported that the number of Lactobacillus was significantly reduced in women with ovarian cancer compared with controls. This change was particularly pronounced in patients with BRCA (1/2) mutations; these mutations seemed to promote the growth of microbial communities dominated by non-Lactobacillus bacteria. Lactobacilli produce lactic acid through glycogen metabolism, and high estrogen levels cause glycogen secretion by vaginal epithelial cells. Widschwendter et al. found that BRCA mutation carriers had higher progesterone levels throughout the menstrual cycle, especially during the luteal phase. High concentrations of progesterone lead to a decrease in vaginal glycogen levels, making the environment unfavorable for the growth of Lactobacilli. However, no clinical studies have confirmed a causal relationship between GM and the risk of OC.

Currently, molecular biology studies have shown that disorders of glycerophospholipid metabolism, sphingolipid metabolism, and glyceride lipid metabolism are important metabolic pathways in the progression of OC. Animal-level studies through liquid chromatography-mass spectrometry (LC-MS) testing of serum from mice with early and advanced HGSOC, respectively, have shown that lipid metabolism disorders such as glycerophospholipid metabolism and sphingolipid metabolism often occur with altered levels of 29 metabolites in the early stages of OC (Huang et al., 2019).

As mentioned above, the specific relevance of GM to the onset of OC has not been fully elucidated, and the mechanism of metabolites between OC and GM is still unknown. Therefore, a thorough study of the relationship between the GM, metabolites, and OC is urgently needed. This study may expand our understanding of the pathogenesis of OC and may provide new biomarkers and therapeutic targets based on multi-omic studies on OC.

Mendelian randomization (MR) utilizes genetic variation as an instrumental variable (IV) for risk factors or exposures and disease occurrence as a clinical outcome to analyze the causal relationship between clinical outcomes and risk factors (Lawlor et al., 2008; Zheng et al., 2017). This approach can avoid confounders and reverse causality in observational studies and allow for more robust causal inferences between exposures and clinical outcomes. In addition, growing evidence has illustrated the value of clinical studies using human genetic information for gut microbial traits, allowing us to employ MR to infer a causal relationship between GM and OC. Herein, employing summary data from genome-wide association studies (GWASs), we conducted MR analysis to explore the causal relationship between GM and OC. Furthermore, we conducted mediation analysis with MR, applying a two-step approach to investigate the total genetic prediction effect of metabolites mediating the GM on the risk of OC, thus guiding the prevention, diagnosis, and treatment of OC.

## 2 Methods

### 2.1 Study design

Single nucleotide polymorphisms (SNPs) were used in this study as IVs to explore the causal relationship between GM and OC. The three criteria listed in the Strengthening the Reporting of Observational Studies in Epidemiology using Mendelian Randomisation (STROBE-MR) checklist (Skrivankova et al., 2021) must be fulfilled. 1) There is a significant association between exposure and each IV. 2) The exposure alone influences each IV’s result. 3) Linkage disequilibrium (LD) reduces the bias by ensuring no confounding factors impact any IV (van Kippersluis and Rietveld, 2018).

We evaluated the causal associations between GM, circulating metabolites, and OC using univariable MR (UVMR) and multivariable MR (MVMR) analysis.

**Figure 1:**
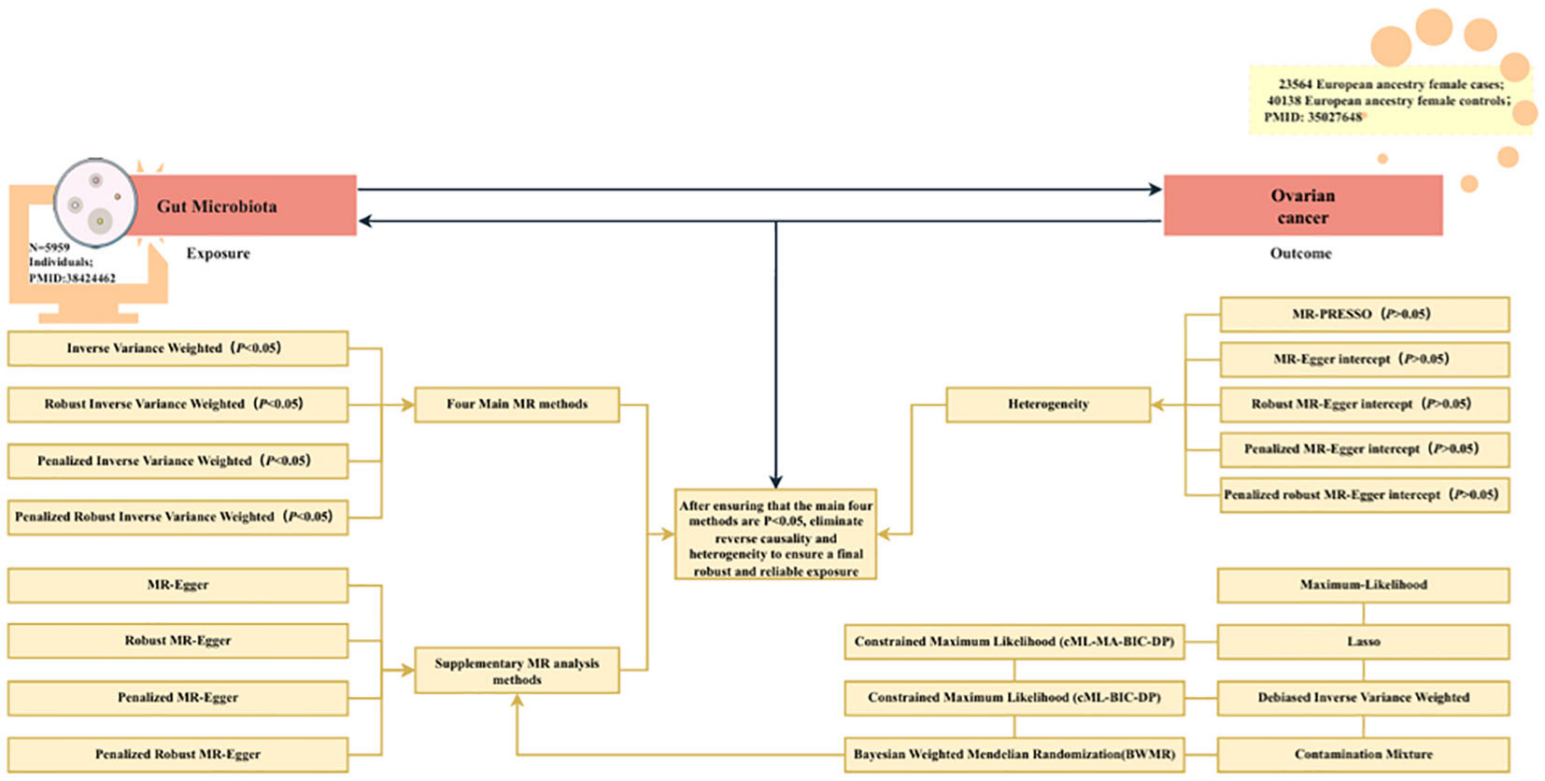
The flowchart of the UVMR study. MR, Mendelian randomization; gut microbiota, used as the exposure; and ovarian cancer, used as the outcome.

**Figure 1** shows the flowchart for the UVMR study. First, the positive UVMR analysis was investigated between GM as the exposure and OC as the outcome; subsequently, the robustness of the causal association between GM and the development of OC was verified using Inverse Variance Weighted (IVW), Robust IVW, Penalized IVW, and Penalized Robust IVW. MR-Egger Intercept, Penalized MR-Egger Intercept, Robust MR-Egger Intercept, and Penalized Robust MR-Egger Intercept were employed to lessen the influence of horizontal pleiotropy. Finally, the MR-PRESSO test was employed to eliminate the abnormal IVs, resulting in the most robust GM following the detection and correction of outliers.

**Figure 2:**
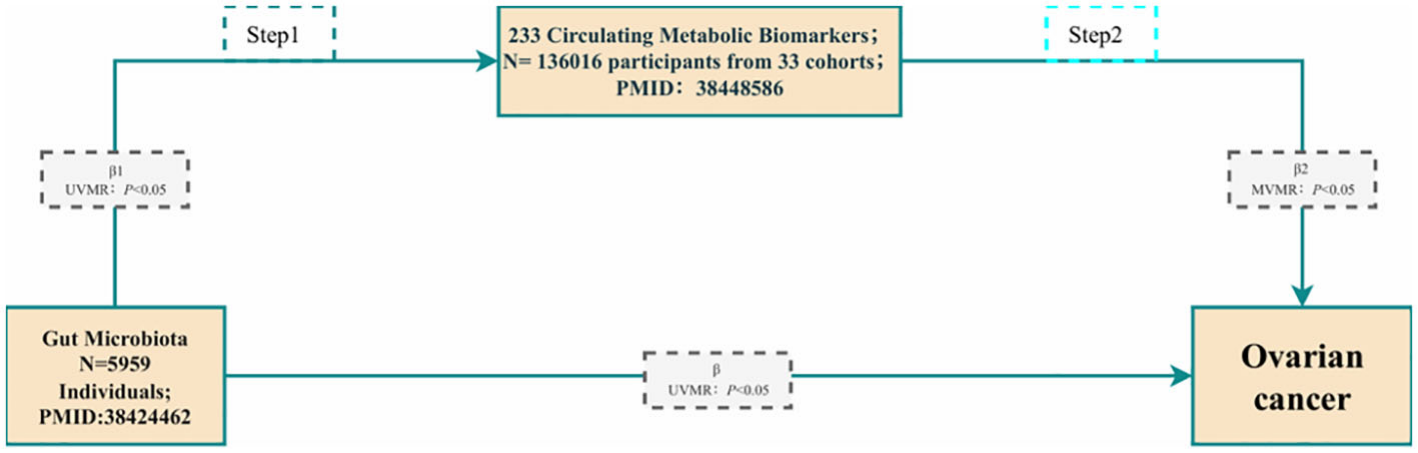
The flowchart of the mediation analysis is based on MVMR. Gut microbiota are used as the exposure; Metabolites are used as the mediator; and Ovarian cancer is used as the outcome.

We further employed a two-step approach based on MVMR to evaluate the total genetic prediction effect of metabolites mediating GM on the risk of OC. The flowchart of mediation analysis based on MVMR is shown in **Figure 2**, with GM as the exposure, metabolites as the mediator, and OC as the outcome. In the first step of the two-step method, routine UVMR analysis of gut microbes and metabolites was performed to obtain β1 (*P* < 0.05). In the second step, MVMR analysis of the positive metabolites, GM, and OC was carried out to yield β2 (*P* < 0.05). In this way, with the UVMR analysis of GM and OC, the direct effect was β-β1 * β2, the mediation effect was β1 * β2/β, and the total effect was β.

### 2.2 Data sources

GWAS data for OC were obtained from the Catalog GWAS database with GCST GCST90016665 (https://www.ebi.ac.uk/gwas/studies/GCST90016665) (Dareng et al., 2022), which included 63,702 participants (23,564 cases and 40,138 controls).

GWAS data for GM were obtained from the study by Qin et al. in 2022 (Qin et al., 2022). This study examined the impact of human genetic variation on the abundance of GM by analyzing data from 5,959 individuals with genotype-matched GM, diet, and health records and identified 567 independent SNP-taxon associations.

The GWAS data for the metabolites were derived from a study published by Karjalainen et al. in 2024 (Karjalainen et al., 2024). With 136,016 participants, a GWAS analysis of 233 circulating metabolic traits revealed over 400 independent loci, of which two-thirds were likely causal genes. This highlighted the significant impact of sample and participant characteristics on genetic associations, revealed the genetic basis of circulating metabolic traits and their impact on complex diseases, and provided an ample data resource for metabolism-disease relationships.

### 2.3 Instrument selection

To ensure the stability of the study data and the accuracy of the results when GM was used as an exposure factor and OC was used as the outcome, we proposed the following requirements for IVs: (a) the significance threshold of P < 1×10^-5^ is applied to IVs related to the GM to ensure genome-wide significance (Cheng et al., 2020); (b) to satisfy the conditions for the MR analysis, we performed an LD analysis based on the European Thousand Person Genome Project, which required R^2^ < 0.001 of IVs, LD = 10000 kb; (c) we assessed the statistical strength of genetic variation as the IVs using the F-statistic to prevent the effect of the allele on the causal relationship between the GM and OC. IVs with an F-statistic of variation ≤ 10 are treated as weak IVs and may bias the analysis results, while an F-statistic > 10 indicates that the IVs are strong. Thus, we excluded IVs with an F-statistic of variation ≤ 10 (Burgess et al., 2017).

Moreover, in the two-step mediation analysis based on MVMR, the IVs of metabolites should meet the following requirements: P < 1×10^-5^, R^2^ < 0.001, LD = 10000 kb. Similarly, IVs with an F-statistic of variation ≤ 10 were excluded.

### 2.4 Statistical analysis

After obtaining the required data from the Catalog GWAS, we conducted an MR analysis to explore the causal relationship between GM and OC. Finally, we applied a two-step mediation analysis with MR to investigate the total genetic prediction effect of metabolite-mediated GM on the risk of OC.

During the MR analysis, we mainly used R (version 4.3.1) with the “Two Sample MR” R package (version 0.5.7) (Mounier and Kutalik, 2023), “Mendelian Randomization” R package (version 0.9.0) and “Bayesian Weighted Mendelian Randomization (BWMR)” R package (version 0.1.1) (Zhao et al., 2020). The R^2^ was used to represent the proportion of the phenotypic variants explained by the SNPs and was calculated by the equation $R^{2}=\frac{2\times \text{β}\times \text{EAF}\times \left(1-\text{EAF}\right)}{2\times {\text{β}}^{2}\times \text{EAF}\times \left(1-\text{EAF}\right)+{\text{SE}}^{2}\times 2\times \text{Sample size}\times \text{EAF}\times \left(1-\text{EAF}\right)}$ . To assess the strength of the IVs; we calculated the F-statistic with the formula $F=\frac{R^{2}\times \left(Samplesize-1-k\right)}{\left(1-R^{2}\right)\times k}$ , where R^2^ was the proportion of phenotypic variation explained by SNPs and k was the number of SNPs included in the tool (Lai et al., 2018). Thresholds with an F-statistic > 10 were generally considered statistically significant, indicating that bias did not affect causal links (Zuber et al., 2020).

In the UVMR study, we first verified the validity of all IVs using the IVW method and obtained the weighted total effect according to the P-value (Brion et al., 2013). To verify the robustness of the conclusions, we used the following three methods to reduce the bias of the causal analysis: (a) using the Robust IVW to reduce the sensitivity of IVs to outliers and strong pleiotropy; (b) using Penalized IVW to adjust for effect estimates of outliers or inconsistency in the data, thus to gain more reliable causal estimates; (c) using Penalized Robust IVW to adjust for outliers in the data and for inconsistent effect estimates, and to reduce the effects of IVs with pleiotropy, thus providing the most rigorous and robust causal estimates. Secondly, the P-value of the MR-Egger intercept was introduced to detect the presence of directional pleiotropy (Burgess et al., 2024). If *P* > 0.05, no obvious directional pleiotropy was considered, which increased the reliability of the causal effect estimate. Meanwhile, to verify the reliability of causal effects in the conclusions, we used four methods to exclude the interference of horizontal pleiotropy: (a) using MR-Egger Intercept to detect the directional pleiotropy of IVs, indicating whether the average effect of pleiotropy differed from zero; (b) using Penalized MR-Egger Intercept, with a penalty term introduced into the MR-Egger Intercept method to reduce the impact of pleiotropic IVs; (c) using the Robust MR-Egger Intercept to adjust the robustness of the MR-Egger Intercept method, thus reducing the effects of outliers and strong pleiotropic IVs; (d) using Penalized Robust MR-Egger Intercept, with both robustness and pleiotropy penalties considered and acting together to improve the accuracy and robustness of causal estimates. Finally, outliers were detected and corrected by removing abnormal IVs using the MR-PRESSO test (Fan et al., 2023), and the results were more reliable when the effect size of IVW was consistent with the sensitivity analysis and *P* < 0.05. We also performed a variety of supplementary MR analyses, including Contamination mixture, maximum likelihood, Debiased IVW, MR-Egger, and BWMR. Although Contamination mixture MR analysis did not remove abnormal IVs, based on the assumption that valid IVs were the largest subset of all IVs, the analysis method would have a more precise causal effect than IVW results (Burgess et al., 2020). The MR analysis method of maximum likelihood is applied to unrelated and related genetic variants. In IVW, if the fixed effect model was incorrect and there was great heterogeneity in the causal effect of different variables, the MR analysis method of maximum likelihood analyzed the existing heterogeneity by random effect model (Burgess et al., 2013). If there were unavoidable weak IVs, we performed MR analysis using the method of Debiased IVW. This approach was robust to many weak IVs and did not require screening (Ye et al., 2021). MR-Egger evaluated whether genetic variation was pleiotropic for results that, on average, differ from zero by targeted pleiotropy tests, causal effect tests, and causal effect estimates, and provided a consistent estimate of causal effect under weaker assumptions (Burgess and Thompson, 2017). BWMR considered the uncertainty of weak effects due to polygenes and detected their outliers through Bayesian Weighted, thus solving the violation of MR assumptions due to polygenes.

In the two-step mediation MR, first, the most robust GM and metabolites were used to perform UVMR analysis to obtain β1; then, the positive mediator (metabolites) determined by the first step was combined with the most robust GM to obtain β2. At this time, with the UVMR analysis of GM and OC, the total effect was β, the mediation effect was β1 * β2, the direct effect was β-β1 * β2, and the mediation effect was β1 * β2/β. In the second step of the two-step MR for MVMR, we used Multivariable IVW to verify the validity of all IVs and generate weighted total effects by judging the magnitude of the P-value.

## 3 Results

### 3.1 Causal effect of the gut microbiota on ovarian cancer

**Figure 3:**
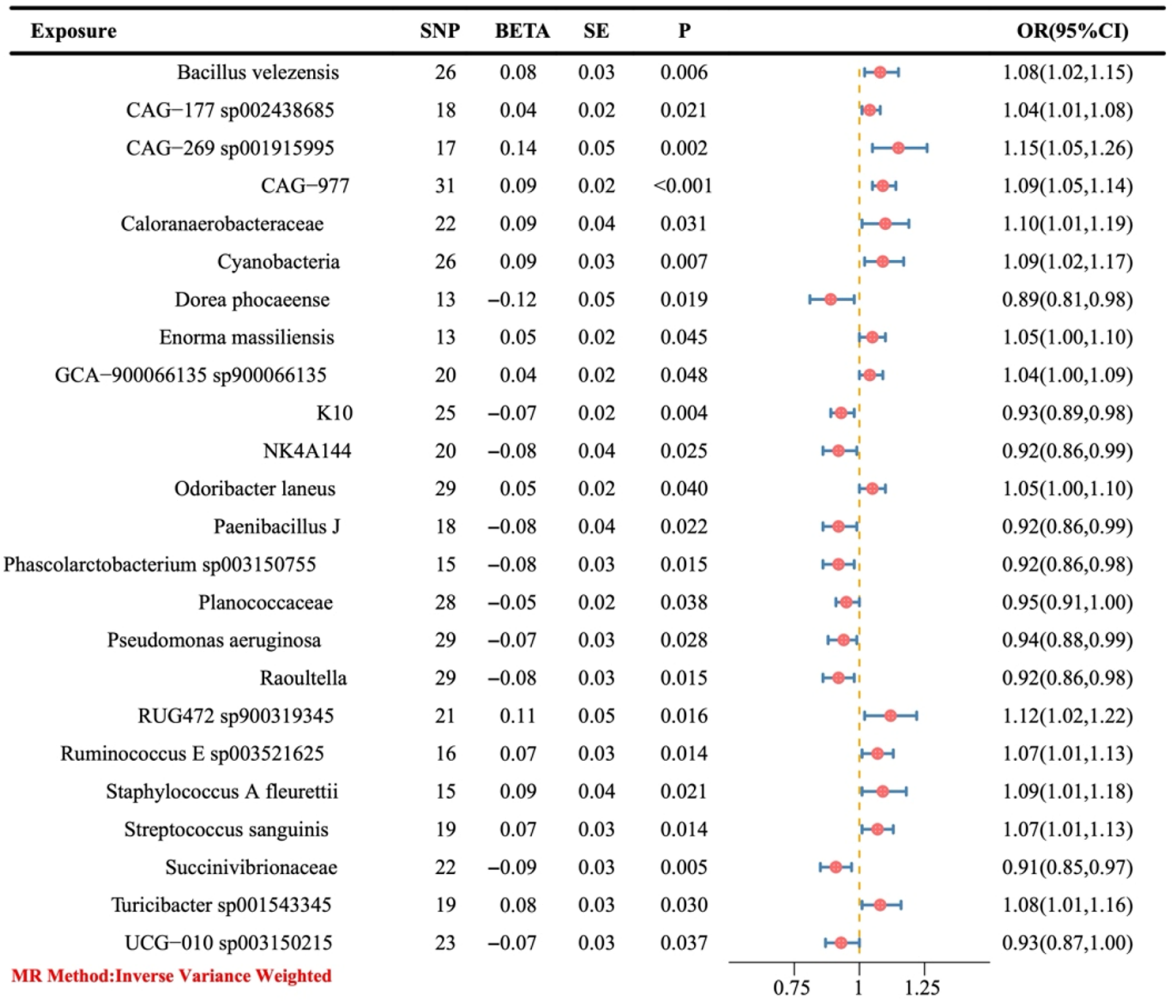
The causal link between gut microbiota and ovarian cancer was assessed using a Mendelian randomization (MR) forest plot. Gut microbiota, used as the exposure; Ovarian cancer, used as the outcome; SE, standard error; CI, confidence interval; SNP, single nucleotide polymorphism; OR, odds ratios.

The preliminary IVW results showed that a total of 24 gut bacteria played a causal role in OC (**Figure 3**), of which the bacteria with a positive correlation were *Dorea phocaeense*, *Succinivibrionaceae*, *Raoultella*, *Phascolarctobacterium sp003150755*, *Paenibacillus J*, *NK4A144*, *K10*, *UCG-010 sp003150215*, *Pseudomonas aeruginosa* and *Planococcaceae* (OR > 1), and the bacteria with a negative correlation were *CAG-177 sp002438685*, *GCA-900066135 sp900066135*, *Enorma massiliensis*, *Odoribacter laneus*, *Ruminococcus E sp003521625*, *Streptococcus sanguinis*, *Turicibacter sp001543345*, and *Bacillus velezensis*, *CAG-977*, *CyanobacteriaStaphylococcus A fleurettii*, *Caloranaerobacteraceae*, *RUG472 sp900319345*, and *CAG-269 sp001915995* (OR > 1).

**Figure 4:**
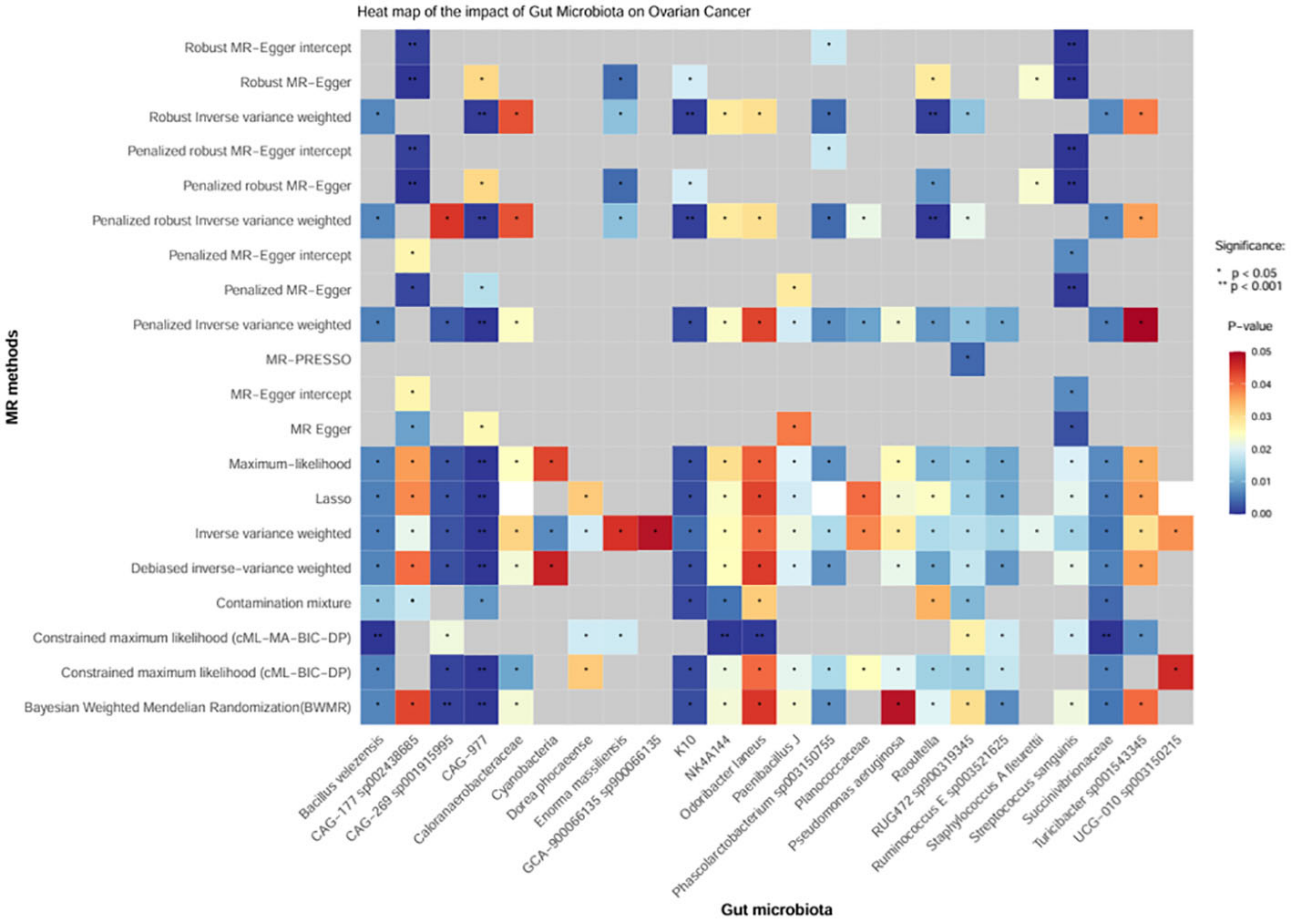
Heatmap of the causal effect of gut microbiota on ovarian cancer. The x-axis represents the 24 positive intestinal bacteria. The y-axis represents various sensitivity analysis methods, horizontal pleiotropy, and removal of abnormal IVs. The change from red to blue indicates P < 0.05; gray represents P > 0.05.

Moreover, we conducted sensitivity analysis, horizontal pleiotropy, and removal of abnormal IVs on the above 24 positive gut bacteria (**Figure 4**), which also confirmed the robustness of the causal association between GM and OC.

### 3.2 Causal effect of the gut microbiota on metabolites

**Figure 5:**
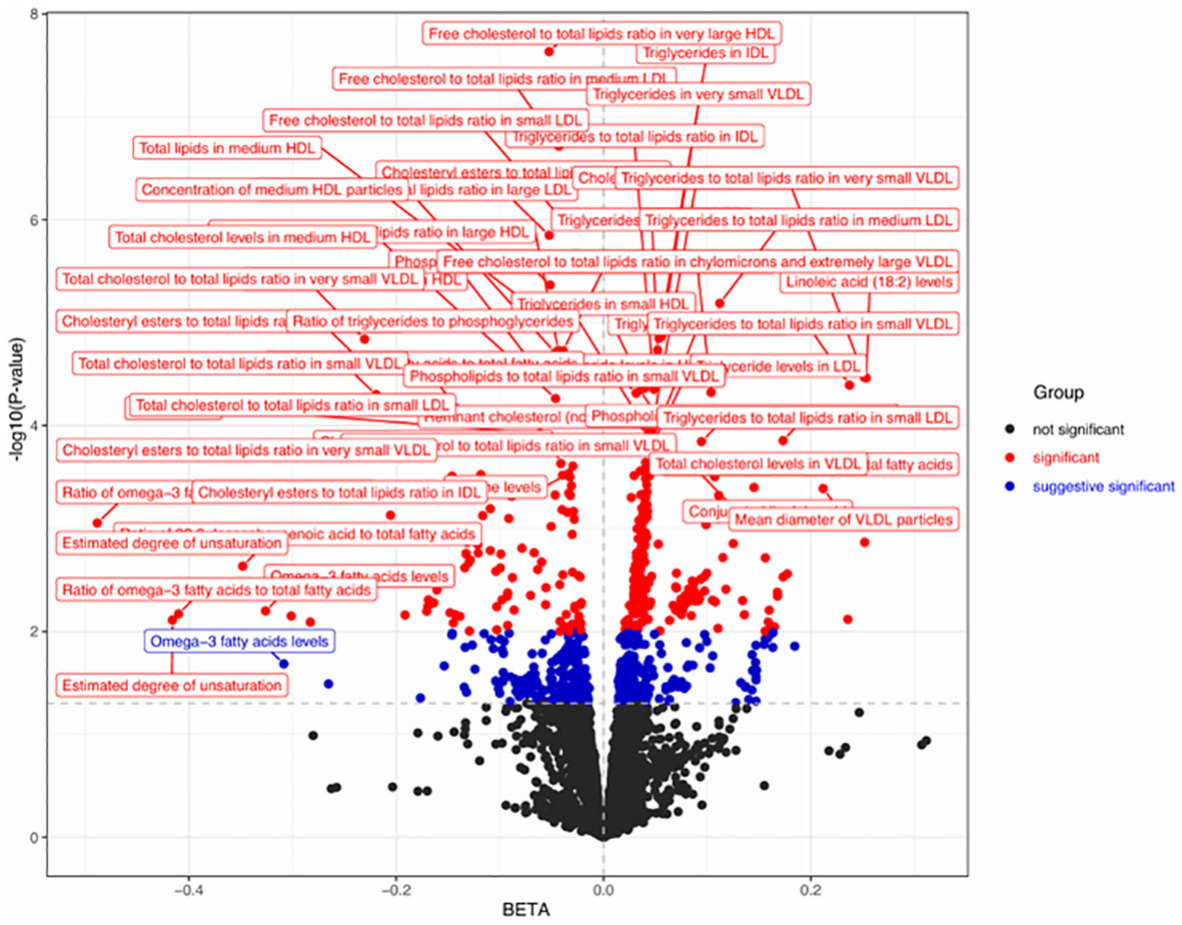
Volcano plot of the causal effect of positive gut bacteria on metabolites. Black dots, P > 0.05; red dots, P < 0.05; blue dots, P<0.001. IDL, intermediate-density lipoprotein; VLDL, very-low-density lipoprotein; HDL, high-density lipoprotein; LDL, low-density lipoprotein.

In MR analysis, we analyzed the above 24 positive gut bacteria with 233 metabolites. As shown in **Figure 5**, 242 positive results were presented as a heatmap (part had been overlapped), which suggested that the above 24 positive gut bacteria may be causally related to the following categories of metabolites. The first category included lipoproteins, such as the ratio of free cholesterol to total lipids in very large high-density lipoprotein (HDL), the ratio of phospholipids in medium HDL, and the total lipids in medium HDL; the second category included lipids, such as levels of linoleic acid (18:2), the ratio of 18:2 linoleic acid to total fatty acids, and the ratio of saturated fatty acids to total fatty acids; the third category included amino acids, such as citrate, glycine, and histidine levels.

### 3.3 Causal effect of metabolites on ovarian cancer

According to the MVMR analysis of the positive gut bacteria and their corresponding positive metabolites together with the outcomes, the metabolites with a causal relationship with OC were obtained. Finally, the mediation effect ratio was calculated. After excluding the mediation effect ratio, which was negative and of P > 0.05, six metabolites that had a causal relationship with OC were gained.

**Figure 6:**
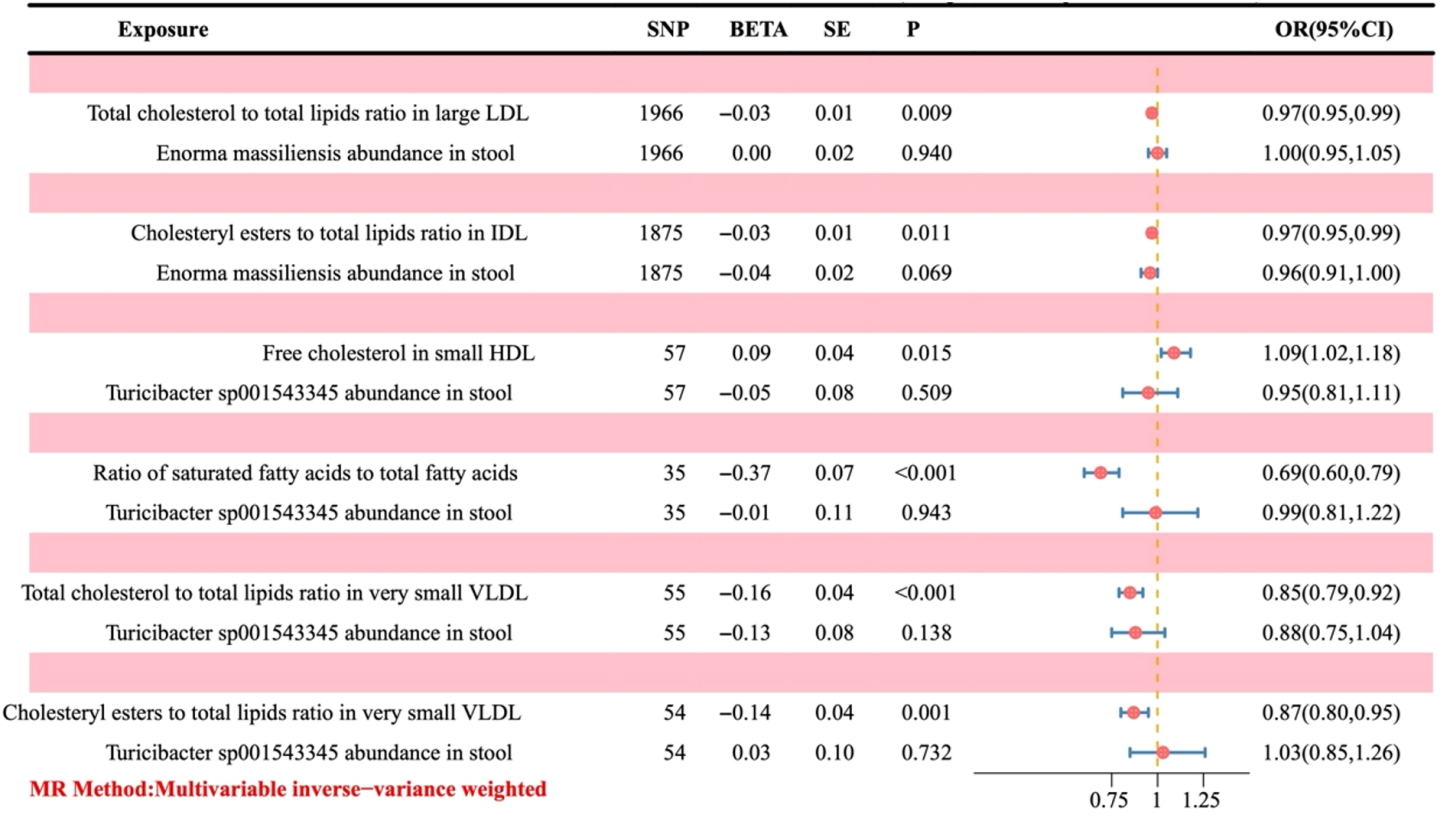
Forest plot of the causal effect of positive gut bacteria and positive metabolites on ovarian cancer. Metabolites are used as the exposure; Ovarian cancer is used as the outcome; OR, odds ratios; CI, confidence interval.

Since OR > 1 is a positive correlation, it indicates that the corresponding metabolite is a risk factor for the outcome. At the same time, OR < 1 is a negative correlation, which indicates that the corresponding metabolite is a protective factor. As illustrated in **Figure 6**, an increase in free cholesterol in small HDL (OR = 1.09, 95% CI 1.02-1.18; P = 0.015) was associated with higher odds of developing OC. Conversely, five other metabolites, including the total cholesterol to total lipids ratio in large low-density lipoprotein (LDL) (OR = 0.97, 95% CI 0.95-0.99; P = 0.009), cholesteryl esters to total lipids ratio in intermediate-density lipoprotein (IDL) (OR = 0.97, 95% CI 0.95-0.99; P = 0.011), the ratio of saturated fatty acids to total fatty acids (OR = 0.69, 95% CI 0.60-0.79; P < 0.001), the total cholesterol to total lipids ratio in very small very-low-density lipoprotein (VLDL) (OR = 0.85, 95% CI 0.79-0.92; P < 0.001), and the cholesteryl esters to total lipids ratio in very small VLDL (OR = 0.87, 95% CI 0.80-0.95; P = 0.001) were linked to decreased odds of developing OC.

### 3.4 Mediation effect of metabolite-mediating genetic predictions of the gut microbiota on ovarian cancer

Based on the causal effect of positive gut bacteria and positive metabolites on the risk of OC, we analyzed the mediation effect of metabolite-mediating genetic predictions of the gut microbiota on the risk of OC (**Table 1**).

**Table 1:** Mediation effect of metabolite-mediating genetic predictions of GM on OC.

Exposure	Mediator	Outcome	Mediation effect ratio	LCI ratio	UCI ratio	Z	P
Enorma massiliensis	Total cholesterol to total lipids ratio in large LDL	Ovarian cancer	1.82%	0.001	0.035	2.13	0.03
Enorma massiliensis	Cholesteryl esters to total lipids ratio in IDL	Ovarian cancer	2.60%	0.001	0.051	2.05	0.04
Turicibacter sp001543345	Free cholesterol in small HDL	Ovarian cancer	11.64%	0.003	0.230	2.00	0.05
Turicibacter sp001543345	Ratio of saturated fatty acids to total fatty acids	Ovarian cancer	69.09%	0.019	1.363	2.02	0.04
Turicibacter sp001543345	Total cholesterol to total lipids ratio in very small VLDL	Ovarian cancer	49.36%	0.139	0.848	2.73	0.01
Turicibacter sp001543345	Cholesteryl esters to total lipids ratio in very small VLDL	Ovarian cancer	35.92%	0.050	0.668	2.28	0.02

In the process of *Enorma massiliensis* acting as a risk factor for OC, the total cholesterol to total lipids ratio in large LDL (mediation effect ratio = 1.82%) and the cholesteryl esters to total lipids ratio in IDL (mediation effect ratio = 2.60%) both mediated the total genetic prediction impact of *Enorma massiliensis* on the risk of OC. This suggests that *Enorma massiliensis* could lower the risk of OC by increasing the total cholesterol to total lipids ratio in large LDL and cholesteryl esters to total lipids ratio in IDL.

*Turicibacter sp001543345* was found to be a risk factor for OC in the following ways: free cholesterol in small HDL (mediation effect ratio = 11.64%), ratio of saturated fatty acids to total fatty acids (69.09%), total cholesterol to total lipids in very small VLDL (mediation effect ratio = 49.36%), and cholesteryl esters to total lipids in very small VLDL (mediation effect ratio = 35.92%). These factors, in turn, mediated the overall genetic prediction impact of *Turicibacter sp001543345* on the risk of OC. These findings suggest that *Turicibacter sp001543345* may lower the risk of OC by reducing free cholesterol in small HDL and increasing the ratio of saturated fatty acids to total fatty acids, total cholesterol to total lipids in very small VLDL, and cholesteryl esters to total lipids in very small VLDL.

## 4 Discussion

In this study, MR analysis found that GM (24 gut bacteria) had a causal relationship with the pathogenesis of OC, among which 10 gut bacteria including *Dorea phocaeense*, *Succinivibrionaceae*, *Raoultella*, *Phascolarctobacterium sp003150755*, *Paenibacillus J*, *NK4A144*, *K10*, *UCG-010 sp003150215*, *Pseudomonas aeruginosa*, and *Planococcaceae* (OR > 1) were risk factors and 14 gut bacteria including *CAG-177 sp002438685*, *GCA-900066135 sp900066135*, *Enorma massiliensis*, *Odoribacter laneus*, *Ruminococcus E sp003521625*, *Streptococcus sanguinis*, *Turicibacter sp001543345*, and *Bacillus velezensis*, *CAG-977*, *CyanobacteriaStaphylococcus A fleurettii*, *Caloranaerobacteraceae*, *RUG472 sp900319345*, and *CAG-269 sp001915995* (OR < 1) were protective factors. Moreover, the robustness of the causal association between GM and OC was verified by IVW, Robust IVW, Penalized IVW, and Penalized Robust IVW.

The UVMR analysis suggested that the above 24 positive gut bacteria were causally related to three categories of metabolites, including lipoproteins, lipids, and amino acids. Furthermore, the MVMR analysis indicated that six metabolites had a causal relationship with OC. The mediation effect of metabolite-mediating genetic predictions of the gut microbiota on the risk of OC suggested that two protective factors (*Enorma massiliensis* and *Turicibacter sp001543345*, OR < 1) could reduce the risk of OC by increasing or decreasing the mediation effect ratio. Specifically*, Enorma massiliensis* can potentially lower the risk of OC by increasing the ratio of total cholesterol to total lipids in large LDL and cholesteryl esters to total lipids in IDL. *Turicibacter sp001543345* can potentially lower the risk of OC by lowering free cholesterol in small HDL and increasing the ratio of saturated fatty acids to total fatty acids and total cholesterol to total lipids ratio in very small VLDL.

Our research results suggested that *Enorma massiliensis* and *Turicibacter sp001543345* may act as protective agents against OC. Notably, *Enorma massiliensis* is a new genus within the family *Coriobacteriaceae, Enorma gen. nov*., and was found in the stool of a 26-year-old woman who had morbid obesity as part of a culturomics study that attempted to cultivate every species of bacteria found in human feces individually (Mishra et al., 2013). This is a rod-shaped, anaerobic, non-endospore-forming, indole-negative bacterium that is Gram-positive. In addition, a study on the gut microbiome of breast cancer patients in Vietnam revealed that patients who experienced a significant delay in diagnosis had higher abundances of Enorma massiliensis (Nguyen et al., 2024). There is very little literature on the study of *Enorma massiliensis*, which also indicates that this genus *Enorma massiliensis* deserves further exploration as a protective factor for the pathogenesis of OC. *Turicibacter sp001543345* is a member of the family genus *Turicibacter*, which can reach relative abundances of 0.5% in the human fecal microbiota (Martínez et al., 2015; Browne et al., 2016). In numerous microbiota community profiling studies, correlations between *Turicibacter* and features of host fat metabolism, including adiposity and dietary lipids, have been revealed (Liu et al., 2016; Jiao et al., 2018; Li et al., 2019; Petersen et al., 2019; Velázquez et al., 2019; Dhakal et al., 2020). A recent study identified genes that allow different strains of *Turicibacter bacteria* to alter host bile acids and lipid metabolism, demonstrating how these bacteria affect host metabolites, including lipids and bile acids. These findings position *Turicibacter bacteria* as modulators of host fat biology (Desorcy-Scherer et al., 2024).

Blood metabolites may be linked to the pathogenesis of OC and can predict survival outcomes, but little is known about the genetics of these metabolites. Previous studies have shown that patients with ovarian tumors have higher cholesterol levels in the ascites. An early report by Helzlsouer et al. indicated a positive correlation between blood cholesterol concentration and ovarian cancer risk. In addition, LDL, as the main transporter of cholesterol, is associated with the aggressiveness of ovarian cancer and poor survival prognosis. In a mouse ID8 ovarian cancer model, mice fed a high-cholesterol diet had accelerated tumor growth compared with the control group. Studies have shown that cholesterol homeostasis disorders may enhance the resistance of ovarian cancer to platinum drugs. At the same time, elevated cholesterol levels in invasive ascites activate LXR α/β nuclear receptors, upregulating multidrug resistance protein 1 (MDR1) and causing ovarian tumor cells to become resistant to cisplatin. Liver cancer cells become resistant to chemotherapy when their mitochondria have a high cholesterol load. It interferes with mitochondrial function, inhibits membrane permeability, and reduces the pro-apoptotic signal cytochrome c release. In addition, cholesterol affects energy metabolism, thereby promoting tumor development. Exogenous cholesterol can alter metabolic pathways, enhance cell proliferation in a manner dependent on the estrogen-related receptor α, elevate oxidative phosphorylation, and activate the tricarboxylic acid cycle (TCA cycle) in breast cancer cells. Studies have found that exogenous cholesterol can enhance aerobic glycolysis in triple-negative breast cancer cell lines. In addition, elevated mitochondrial cholesterol load promotes hexokinase transfer to mitochondria and may augment aerobic glycolysis in cancer cells. In this study, we analyzed the mediation effect of metabolite-mediating genetic predictions of the gut microbiota on the risk of OC based on the causal effect of 24 positive gut bacteria and 6 positive metabolites on the risk of OC, providing insights into the positive gut bacteria could affect the risk of OC by decreasing or increasing the mediation effect ratio of corresponding metabolites. This study evaluated the causal relationship between GM and the pathogenesis of OC based on MR analysis and confirmed the association of GM with the pathogenesis of OC. The advantages of this study mainly included the following aspects: first, MR could infer the causal association of exposure to the outcome and not be affected by confounding factors; second, GWAS data acquisition based on large population samples in this study improved the reliability of the results; finally, multiple methods were applied to reduce the bias of the causal analysis, thus ensuring the confidence and robustness of the results. However, the present study also had some limitations. On the one hand, the data set used in the study may have some unknown confounders that would impact the results. On the other hand, the GM GWAS data contained multiple populations, but mainly European populations, and the OC GWAS data was also a European population, so the universality of the conclusion needs to be further confirmed.

In conclusion, this study explored the causality of GM, metabolites, and risk of OC and revealed the mediation effect of metabolite-mediating genetic predictions of GM on OC. Therefore, this study could reference GM-based control measures against OC. On this basis, in the future, it is necessary to explore further the mechanism of causality of specific gut bacteria, specific metabolites, and the risk of OC and find new strategies for preventing and treating OC with specific gut bacteria and metabolites.

## Data Availability

The original contributions presented in the study are included in the article/supplementary material. Further inquiries can be directed to the corresponding author.
